# Improving program targeting to combat early-life mortality by identifying high-risk births: an application to India

**DOI:** 10.1186/s12963-018-0172-6

**Published:** 2018-08-23

**Authors:** Antonio P. Ramos, Robert E. Weiss, Jody S. Heymann

**Affiliations:** 1 Department of Biostatistics, Fielding School of Public Health, UCLA, Los Angeles, CA USA; 2 WORLD Policy Analysis Center, Fielding School of Public Health, UCLA, Los Angeles, CA USA

**Keywords:** Early-life mortality, Program targeting, Risk factors, Bayesian hierarchical model

## Abstract

**Background:**

It is widely recognized that there are multiple risk factors for early-life mortality. In practice most interventions to curb early-life mortality target births based on a single risk factor, such as poverty. However, most premature deaths are not from the targeted group. Thus interventions target many births that are at not at high risk and miss many births at high risk.

**Methods:**

Using data from the second wave of Demographic and Health Surveys from India and a hierarchical Bayesian model, we estimate infant mortality risk for 73.320 infants in India as a function of 4 risk factors. We show how this information can be used to improve program targeting. We compare our novel approach against common programs that target groups based on a single risk factor.

**Results:**

A conventional approach that targets mothers in the lowest quintile of income correctly identifies only 30% of infant deaths. By contrast, using four risk factors simultaneously we identify a group of births of the same size that includes 57% of all deaths. Using the 2012 census to translate these percentages into numbers, there were 25.642.200 births in 2012 and 4.4% died before the age of one. Our approach correctly identifies 643.106 of 1.128.257 infant deaths while poverty only identifies 338.477 infant deaths.

**Conclusion:**

Our approach considerably improves program targeting by identifying more infant deaths than the usual approach that targets births based on a single risk factor. This leads to more efficient program targeting. This is particularly useful in developing countries, where resources are lacking and needs are high.

**Electronic supplementary material:**

The online version of this article (10.1186/s12963-018-0172-6) contains supplementary material, which is available to authorized users.

## Background

Inequality in early-life mortality (ELM) is a fundamental dimension of social inequality. For example, the Millennium Development Goals (MDG) include reduction in ELM among its goals [1, 2]. For the 195 countries with available data, 68% failed to achieve the reductions in ELM established by Goal 4 by 2015 [3, 4]. Earlier studies have suggested that the MDG would be difficult to achieve precisely because of high levels of inequality in ELM that plague many countries [5–7].

Within countries, inequality in ELM is usually defined in terms of differences in average mortality across different levels of a single risk factor. For example, disparities have been documented across income groups, race and ethnic groups and place of residency [8–13]. Current ELM health policies often target births based on a single risk factor, most commonly poverty, for simplicity and because infants in poor households have higher average mortality rates compared to infants in richer households [6, 8, 14–18]. Essentially, this approach uses membership in a group defined by a single risk factor as a proxy for being a birth at a higher risk of premature death.

The most common interventions that target births based on poverty are perhaps Cash Transfer Programs (CTP), now widely implemented in many low- and middle-income countries [19–21]. However, there are many other types of child health interventions that target births from poor families [22–24]. While births from the poorest families have higher mortality rates than other births, targeting births based solely on poverty – or based on any other risk factor – ignores within-group heterogeneity, where births from the same group may have very different mortality risks [5, 25–27]. Targeting groups that are highly heterogeneous in mortality risk is inefficient for program targeting because it allocates resources to lower-risk births not in need of program resources. In particular, groups with highest average mortality are not exclusively populated by high-risk births, and high-risk births exist in other groups despite lower average mortality. As an example, our own calculation using data from the Demographic and Health Surveys for 50 countries from 1970 to 2005 shows that the poorest 20% in each country only contains 30% of all infant deaths from that period.

We develop methodology that improves program targeting by simultaneously combining information from several risk factors. We also set out allocation rules to guide program targeting. We illustrate our approach with a statistical analysis of data from India. We include risk factors identified in the literature that are potentially available for policymakers in other high-mortality and low-resource settings [28, 29]. Further, we purposely have chosen only a few risk factors to demonstrate the power of our approach even when limited information is available, and to facilitate actual policy interventions as several risk factors can make targeting more complex. Our objective is to elucidate methods useful for policymakers who will design policy interventions in high-mortality and low-resource settings.

## Methods

### Data source

We analyze data on 73,320 births from the second wave of the National Family Health Surveys (NFHS) in India, also known as the Demography and Health Surveys (DHS), (https://dhsprogram.com/). This is a nationally representative survey, conducted in 1998–1999, and the last wave in which participants’ district level was reported. Because geographic location is an important risk factor, we use this wave rather than the most recent to illustrate our methodology. We use infant mortality as our outcome and analyze the last five years of births.

In our statistical model we use districts (436), which are the districts in which the infant was born; wealth quintiles (five categories) of the household in which the mother lived at the time of the survey; maternal education (four categories), which is the highest attained level of education of the mother at the time of the interview; and the age of the mother at the birth of the infant. Table 1 summarizes the data.

**Table 1:** Summary statistics for the births in our data set

Risk factor	Number of births
Maternal age	
< 18	9391
19–35	60,201
> 35	3728
Wealth index	
Lowest quintile	14,951
Second quintile	14,492
Middle quintile	15,159
Fourth quintile	15,753
Highest quintile	12,965
Maternal education	
No education	40,341
Primary	11,941
Secondary	15,808
Higher	5230

Sample size is 73,320. The number of districts is 436

### Statistical analysis

Our analysis is a two-step procedure. As mortality risk is a latent variable, we estimate it for each birth in our data using a statistical model. Second, we classify births into small sub-groups to identify groups for program targeting.

We use a Bayesian hierarchical model to predict mortality risk for each birth. The outcome Y_i_ is whether the fifth birth survived to the age of 1 or not. The model predicts _i_, the underlying mortality risk for birth i. We include as predictors the main effects for maternal age, maternal education, household wealth, and district and all two-, three-, and four-way interactions. The main effects and interactions in the regression model are modeled as either fixed or random effects. If a particular effect, either main or interaction, has more than 20 unique levels, we include it as random effect. For example, the main effect of district and the 3-way interaction of age x education x wealth are modeled as random effects. Otherwise, the effects are treated as fixed effects. For random effects, we estimate the prior variance of the random effects. For the fixed effect, we take its prior variance x a known value. We use R and the package MCMCglmm to t our statistical models [30, 31].

After estimating mortality risk, we cross-classify births into small subgroups by geographical location and risk factors. We rank subgroups by average mortality risk, from highest to the lowest mortality risk. The conventional approach targets infants in the lowest wealth quintile, which comprises 20% of the births. Thus, to construct a comparable intervention group with our method, we allocate subgroups, starting with the highest mortality subgroups, until the total percentage adds up to 20% of all births. We consider three scenarios: 1) district: policymakers are allowed to flexibly target different subpopulations in each district; 2) state: target subpopulations can vary by state but all districts within the same state must target the same groups; 3) national: the same groups must be targeted for the entire country. District is the preferred scenario as it allows greatest flexibility in program targeting. However, we also consider state and national scenarios to illustrate the usefulness of our approach even when policy is subject to constraints. Details of our allocation mechanism can be found in the Additional file 1.

## Results

**Fig. 1:**
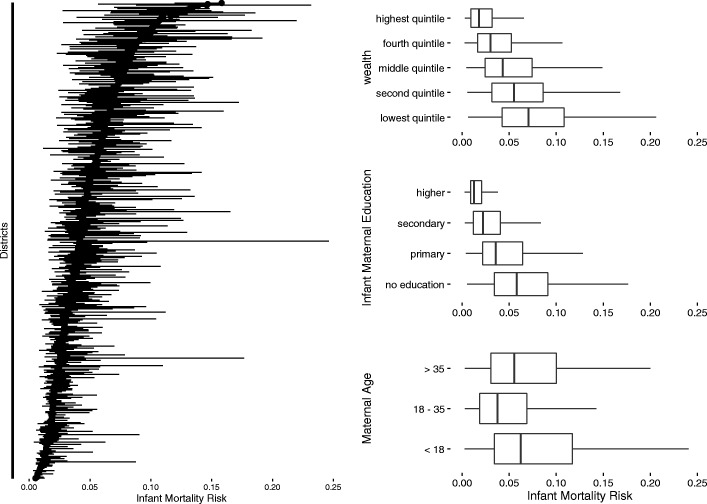
Distribution of estimated infant mortality risk in India, 1993-1998, by categories of risk factor. The three graphs on the right are box plots for, from top to bottom, wealth quintiles, four levels of maternal education, and three categories of maternal age. On the left panel is a plot of the range of the estimated infant mortality risk by district, where districts are ordered by median mortality risk and the horizontal lines extend from the 25% to the 75% of the distribution. These figures show considerable variation in mortality risk in groups defined by levels of a single risk factor

We evaluate first how much variation in mortality risk exists within and between groups defined by levels of single risk factors. Figure 1 on the right displays dot plots of estimated infant mortality risk by risk factors: maternal education, wealth, and maternal age. Districts is a box plot on the left.

Lower income quintiles have higher average mortality risk than the wealthier quantiles. The average risk for the poorest quintile is 8%. However, 15% of all births in other richer quintiles have mortality risk higher than the average risk for the poorest quintile. Maternal education categories exhibit substantial overlap in mortality risk. Average mortality risk for births from women with no education is 6%. However, 27% of infants of mothers from the other educational categories have mortality risk higher than 6%. On the left of Fig. 1, we use a dot plot to display mortality risk by district. Each horizontal line represents a district, and districts are ordered by median risk within district. For each district, the solid line shows the interquartile range of mortality risk, extending from the 25th percentile to the 75th percentile. District median levels of mortality risk range from nearly zero up to 15%. Generally, districts with lower median mortality risk also have lower within-district variation. Within districts, the width of the interquartile range varies from zero up to 10% for districts with wide variation in mortality risk. Thus, districts vary greatly in their level of inequality. Overall, these results suggest that groups defined by levels of a single risk factor have large variability in mortality risk and this makes program targeting based on a single risk factor inefficient.

Table 2 compares the traditional single risk factor program targeting approach to our approach. Using poverty (lowest wealth quintile) as a risk factor to predict mortality, the traditional approach correctly identifies 30% of all deaths. However, our multifactor approach correctly identifies 57% (district), 40% (state) and 38% (national) of all deaths.

**Table 2:** Classi_cation rates comparing statistical approaches against the convetional approach that target poverty (lowest quintile)

Target group	% of correctly classified deaths
Poorest quintile	30%
District	57%
State	40%
National	38%

**Fig. 2:**
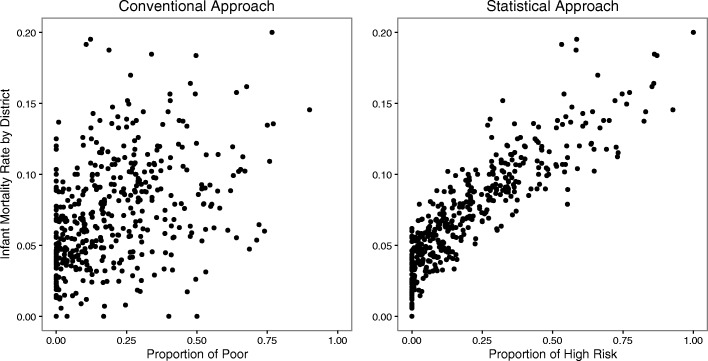
District level comparisons: comparing the fraction of high risk births with infant mortality rates by district. In both panels, district mor- tality rates are plotted on the y-axis. In the left panel the x-axis is the proportion of births from poor families, those in the lowest wealth quintile. In the right panel the x-axis is the fraction of high risk births (20% highest risk births) identified by our model. Our estimates based on the statistical model match more closely the actual mortality rates than using the lowest quintile as a proxy for mortality risk

**Fig. 3:**
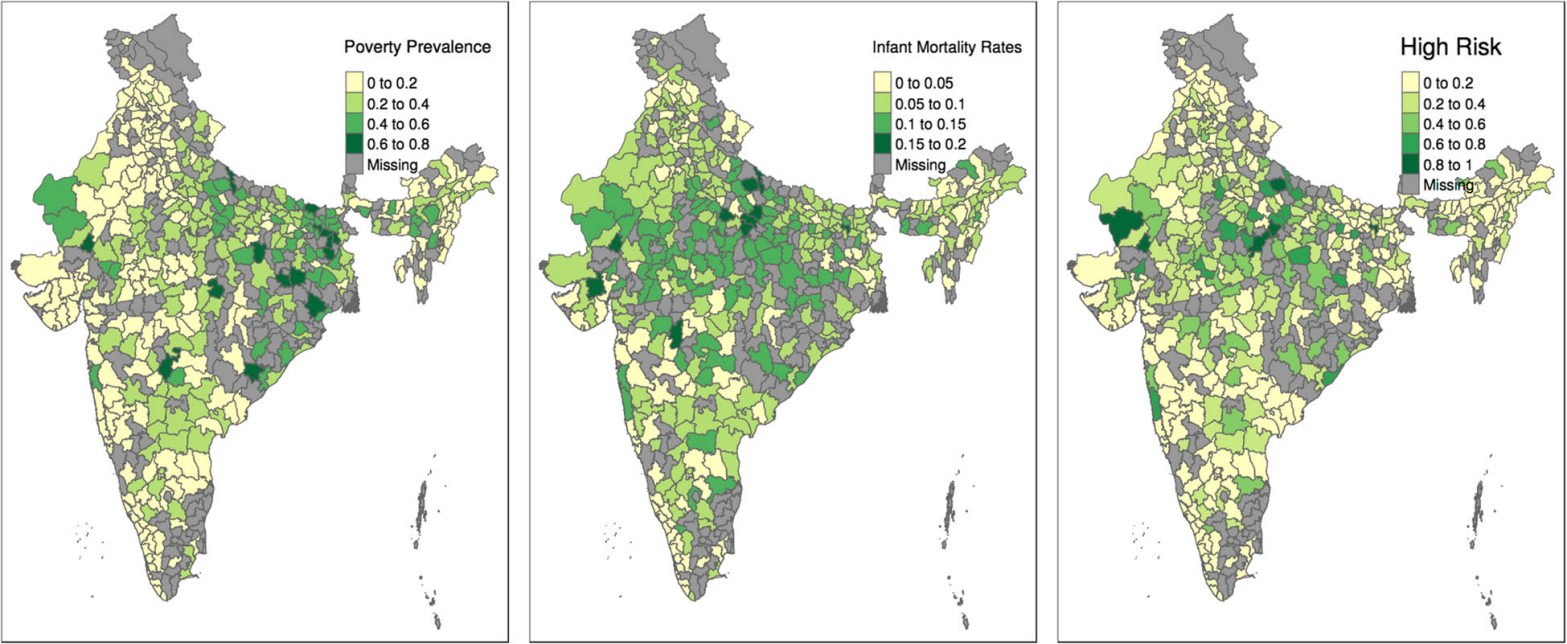
Maps contrasting high risk births by districts: births from the poorest 20 % families, from the 20% higher risk from our statistical model, and actual infant mortality rates

Figure 2 illustrates the efficiency gains from our multi-factor approach against the conventional approach by looking at the data at the district level. Each point in a dot plot is a district, where the y-axis represents the actual proportion of deaths in that district and the x-axis represents the estimated proportion of high-risk births by districts, for each approach. In the left graph, the relationship is weak between infant mortality rates by district and the proportion of deaths in the lowest wealth quintile. By contrast, the estimated mortality risk by district from our statistical model predicts actual deaths at the district level much more precisely. The maps in Fig. 3 contrast poverty (births from the poorest 20% of mothers) and the 20% of highest-risk infants based on our statistical model and infant mortality, all by district.

## Discussion

Our results show that poverty alone is an inefficient guide for program targeting. In contrast, combining information from multiple risk factors significantly improves program targeting efficiency. This is an important finding because program targeting usually uses information from a single risk factor, such as poverty or maternal education, to define the targets [8, 14, 32]. The typical assumption is that because a given group has the highest average mortality risk, most births with high mortality risk belong to that group. However, we have shown that (1) not all births from the highest risk group are at higher mortality risk, and (2) high-risk births also exist in significant numbers across other groups. In agreement with previous literature, geographic location is a particularly important predictor for India, and the relative importance of each risk factor and their interactions varies across districts [28, 29]. Thus, allowing geographic flexibility in designing program interventions can greatly improve policymakers’ ability to target.

Although we used data from India in 1998, we believe our findings are directly applicable to other high-mortality, low-resource settings. First, ELM conditions in India in 1998 are similar to that of many developing countries today. For example, infant mortality rate in 1988 in India was 65 deaths per thousand births, which is lower than 2016 figures for countries like Sierra Leone (83 deaths per thousand births), Central Africa Republic (90 deaths per thousand births), Democratic Republic of Congo (72 deaths per thousand births), Somalia (83 deaths per thousand births), and many other countries. In addition, using data from 100 DHS surveys from 50 lower-middle income countries (LMICs) from 1970 to 2010, we find that the distribution of ELM across the spectrum of income is similar to that in India. Births from the 20% poorest families account for approximately 30% of all deaths, while roughly 70% of all infant deaths occur outside the 20% poorest families. Thus, it seems that targeting based only on income in most LMICs will generate program targeting inefficiencies that our methodology can improve upon. The relative importance of different risk factors and the nuances of program targeting are different in different countries, but these differences can be accommodated within our methodology.

Second, many interventions in global health target the poor. The most common interventions are perhaps Cash Transfer Programs (CTP), currently widely implemented in many LMICs. Many of these programs also intervene in infant and child health [19, 20]. For example, in Burkina Faso, families enrolled in conditional cash transfer schemes were required to obtain quarterly child growth monitoring at local health clinics for all children under 60 months of age [21]. The famous randomized controlled trial (RCT) “Lentils for Vaccines” in India targeted the poor, as do most RCTs that aim to increase vaccine uptake, good nutrition, or general child health [22]. Many anti-poverty programs target child health, such as in Peru [23]. Finally, it is often recommended that poor births should be the target of global health interventions [24].

It is important to stress that our approach is not a critique of programs that target the poor, as we believe that the poor are targeted for good reasons. However, we also believe that our approach can increase program efficiency in high-mortality, low-resource settings.

Finally, even if a given program already targets births based on multiple risk factors (e.g. poor families from rural areas) our approach can still be useful in increasing program efficiency. This is the case because our approach allows policymakers to combine multiple risk factors simultaneously to estimate mortality risk more accurately. Using our approach, policymakers do not need to decide ex-ante which factors define higher-risk groups. Instead, policymakers can use our approach to let the data decide which demographics have higher mortality risk to guide their targeting decisions.

In this paper we explore the role of a few risk factors in predicting early mortality, which were purposely chosen to produce implementable policy recommendations. However, there are a number of other variables that have been linked to ELM, such as sanitation, water supply, birth spacing, and so on. These data are routinely collected by health surveys and governments. Future studies should explore these factors to establish which ones are most useful in improving program targeting. Flexible statistical models including Bayesian semi-parametric and machine learning models can handle a large number of risk factors and interactions, allowing us to investigate numerous risk factors simultaneously [33, 34]. These statistical methods have proven useful in many contexts where predicting a rare event was a key scientific objective. However, the challenge with more data and more complex models is to make clear policy recommendations.

Finally, our approach can be applied to other health outcomes. For example, maternal mortality is closely related to ELM and is a major health problem in LMICs, where more than 289,000 women die during pregnancy and childbirth from preventable causes every year [35, 36]. Among the 122 million women who have a live birth annually, 10% suffer complications and disability. Developing countries account for 99% of global maternal deaths, the majority of which are in sub-Saharan Africa and southern Asia. Programs usually target mothers based on a single or a few risk factors [35]. Our methodology adapts naturally to improve program targeting by exploring combinations of risk factors for maternal mortality and health.

In this paper we illustrate the usefulness of our approach using data from India. To actually implement policies in India or any other country, policymakers need data that are representative of the target population. Thus, the actual implementation of our methods are limited by current or future data collection. A second limitation is related to making practical policy recommendations from more complex statistical models that use additional risk factors simultaneously. These models can potentially identify mortality risk more accurately than those that use fewer risk factors. However, policy recommendations from these models may not necessarily be feasible for policymakers to implement. Policymakers might not be allowed to target certain demographic groups due to political, cultural, or historical reasons, even if they are at higher risk of mortality. Moreover, targeting certain groups can be difficult for logistical reasons. For example, if high-risk births are geographically spread out in such a way that each location has only few high-risk births, it may be costly for policymakers to target births under these circumstances.

## Conclusion

We propose new program targeting methodology that uses information from multiple risk factors simultaneously and a statistical model to better define the target high-risk population. We use India to illustrate our approach, showing that it leads to significant improvements in program targeting over the conventional targeting approach that equates high-risk with the worst level of a single risk factor. We estimate the unobserved mortality risk for each infant in our data, and, using these estimates, we show that the distribution of the mortality risk is highly variable across groups defined by a single factor. This suggests that groups defined by a single risk factor are very heterogeneous in terms of mortality risk, which leads to inefficient targeting. We contrast our approach with the conventional approach to more efficiently identify infant deaths. We compare the 20% poorest births with the 20% highest risk infants. Using poverty as a single risk factor correctly identifies 30% of deaths, while our statistical model correctly identifies 57%. Using India data from 2010 to translate these percentages into numbers, the statistical models correctly identify 506,409 more deaths than the conventional approach.

This study supports the view that monitoring inequality in ELM across births is useful for policy purposes, answering initial skepticism [16, 25, 37]. Our study suggests that looking at national averages is not enough to achieve progress in early-life mortality [5, 7, 25, 38]. Our methodology can be used by policymakers in high-mortality, low-resource settings to improve program intervention and thus help countries to reduce inequality in ELM and meet the Sustainable Development Goals.

## Additional file

Additional file 1: Supplementary Materials. (PDF 116 kb)
