# Supplementary material for: Improving program targeting to combat early-life mortality by identifying high-risk births: an application to India
Source: Popul Health Metr. 2018 Aug 23;16:15. doi: 10.1186/s12963-018-0172-6 (PMC6108144; doi:10.1186/s12963-018-0172-6)
Supplement: Supplementary file 1 — Supplementary Materials. (PDF 116 kb) [file 12963_2018_172_MOESM1_ESM.pdf]

Supplementary Materials

Antonio P. Ramos[1], Robert E. Weiss[2], and Jody S. Heymann[3]

[1]PhD, World Policy Analysis Center and Department of Biostatistics, Fielding School of Public Health, UCLA, California, USA, Corresponding Author: tomramos@ucla.edu

[2]PhD, Department of Biostatistics, Fielding School of Public Health, UCLA, Los Angeles, California, USA, robweiss@ucla.edu

[3]PhD, World Policy Analysis Center, Fielding School of Public Health, UCLA, Los Angeles, California, USA, jody.heymann@ph.ucla.edu

Introduction

Our approach has three steps. In step one, we estimate each infant's mortality risk using a Bayesian hierarchical model. Next, we cross-classify births into cells based on their risk factors combinations. Finally, we select cells with the highest estimated mortality risk to target births with interventions. We gave details of these steps in this document.

Statistical model

We use a Bayesian hierarchical logistic regression model to predict the probability of mortality for an infant by one year after birth. The model includes the following risk factors listed in the main text: age of the mother at birth classified into three categories: under 19 years old, from 19 up to 35 years old, and older than 35 years old; the highest level of education achieved by the mother classified into four categories: no education, primary education, secondary education and higher education; wealth categorized into five wealth quintiles; and the 436 districts. We include all main effects as well as 2-way, 3-way, and 4-way interactions. Main effects and interactions are modeled as either fixed or random effects. If a particular effect, either main or interaction, has more than 20 unique levels, it is included as a random effect. For example, the main effect of district, and the 3-way interaction of

age by education by wealth are treated as random effects; otherwise effects are treated as fixed effects. The intercept was given a Gaussian prior with mean logit(0.1) and unit variance, where $\text{logit}(p) = \log((p)/(1-p))$. All other fixed effects were given standard Gaussian $(0, 1)$ priors. All random effects were given mean zero Gaussian priors with unknown variances. For the variances, we use an inverse-gamma (ψ, ν), for the variance of each effect, with $\psi = 10 - k$, where k is the order of the interaction and $\nu = 10$. This specification shrinks the random effects more towards zero for higher order interaction terms.

Models are fit using the `MCMCglmm` package in the `R` statistical environment.[1,2] We ran two chains for each model, using 230,000 simulations for each chain, letting the first 30,000 interactions be burn-in and then thinning every 25, giving 8,000 posterior samples from each chain. We assessed MCMC convergence using standard graphical and statistical procedures and convergence was deemed satisfactory.

Selection of the target population

We use the risk factor combinations to cross-classify infants mortality risk π_i into cells $c = 1, \ldots, C$ that are amenable to program targeting. Allowable risk factor combinations are dependent on the particular allocation

mechanism. We consider three different scenarios where policy makers face increasing constraints in their flexibility to institute policy. However, our approach is similar in all scenarios. First, we calculate the average mortality risk in each cell $\bar{\Pi}_c = \frac{\sum_{i=1}^{n_c} \pi_i}{n_c}$ where n_c is the number of births in the cell c. The conventional approach targets infants in the lowest wealth quintile, which comprises 20% of the births. Thus to construct a comparable intervention group with our method, we allocate births, starting with the highest mortality cells, until the total percentage adds up to 20% of all births.

District: Policymakers are allowed to select diferent risk factor combinations in different districts.

Under this scenario, we use the following risk factor combinations to group births into cells: maternal age (3 categories), maternal education (4 categories), wealth (5 categories), and 436 districts. Thus our sample of births is divided into $3 * 4 * 5 * 436 = 26,160$ cells. The highest efficiency gains occur in this scenario because it allows the highest flexibility in selecting the target population.

National: Policymakers need to pick the same risk factor combinations nationally.

In this scenario, the restriction is that risk factor combinations must be the same across all districts in the country. Thus we cross-classify our births into $3 * 4 * 5 = 60$ cells. In this scenario policy makers face the greatest constraints in their ability to implement program targeting.

States: Policymakers may select different risk factor combinations by state but within states the combinations need to be the same.

Policy makers may target births with different risk factors in different states but, within a state, all districts target births with identical risk factors. Thus births are cross-classified into 3*4*5*27=1620 cells. Different states will contribute different percentages of births to the target population. The proportion of births from each state will be selected to maximize the mortality risk of the final national sample. Under this scenario, policy makers have more flexibility than in the National scenario but less flexibility than in the Districts scenario.

Extensions and conclusions

Our approach can be extended in a number of ways. First, the current model uses only four risk factors. However, demographic and health surveys collect a vast amount of information that can be potentially be useful in estimation mortality risk. Further work needs to be done to identify the number and types of risk factors combinations that optimize the results. The allocation mechanism can also be further developed to ensure optimal results under complex sets of constraints.

Our approach suggests that the more flexibility policymakers have in selecting the target population, the greater the program targeting gains. Most program targeting operates under constraints. Even under constraints, considering multiple risk factors increases the value of the program.

References

1 Hadfield JD. MCMC Methods for Multi-Response Generalized Linear Mixed Models: The MCMCglmm R Package. Journal of Statistical Software. 2010;33(2):1–22.

2 R Core Team. R: A Language and Environment for Statistical Computing. Vienna, Austria;.
